# Psoriasis may increase the risk of idiopathic pulmonary fibrosis: a two-sample Mendelian randomization study

**DOI:** 10.1186/s12931-024-02721-5

**Published:** 2024-02-26

**Authors:** Lingli Chen, Yujie Wei, Mengjin Hu, Yile Liu, Xiangrong Zheng

**Affiliations:** 1 Department of Pediatrics, Xiangya Hospital, Central South University, Hunan, 410008 China; 2 Key Laboratory of Digestive System Tumors of Gansu Province, Second Hospital of Lanzhou University, Lanzhou, 730000 China; 3 Xuanwu Hospital, Capital Medical University, Beijing, 100053 China

**Keywords:** Psoriasis, Idiopathic pulmonary fibrosis, Epidemiology, Mendelian randomization

## Abstract

**Background:**

Although some studies have indicated that Psoriasis could contribute to the risk of idiopathic pulmonary fibrosis (IPF), no study has reported a clear causal association between them. Our aim was to explore the potential relationship between Psoriasis and IPF using Mendelian randomization (MR) design.

**Methods:**

To explore a causal association between Psoriasis and IPF, we used genetic instruments from the largest available genome-wide association study (GWAS) of European ancestry, including psoriasis (5314 cases, 457,619 controls) and IPF (1028 cases, 196,986 controls). Our main analyses were conducted by inverse-variance weighted (IVW) method with random-effects model, with the other complementary four analyses: weighted median method, weighted mode, multivariable MR and MR-Egger approach.

**Results:**

The results of IVW methods demonstrated that genetically predicted psoriasis was significantly associated with higher odds of IPF, with an odds ratio (OR) of 1.09 (95%CI, 1.01–1.18; P = 0.02). Weighted median method, weighted mode and multivariable MR also demonstrated directionally similar results (P < 0.05), while the MR-Egger regression did not reveal the impact of psoriasis on IPF (OR = 1.09, 95%CI, 0.98–1.21; P = 0.11). In addition, both funnel plots and MR-Egger intercepts indicated no directional pleiotropic effects between psoriasis and IPF.

**Conclusions:**

Our study provided potential evidence between genetically predicted psoriasis and IPF, which suggests that understanding the mutual risk factors between psoriasis and IPF can facilitate the clinical management of both diseases.

## Introduction

Psoriasis is an autoimmune, chronic inflammatory diseases of skin which usually manifested as raised, well-demarcated, erythematous oval plaques with adherent silvery scales, affecting approximately 2% of the population in the world [1]. Findings from immunological and genetic studies have highlighted the crucial role of crosstalk between the innate and adaptive immune systems and the link to genetics in the development of psoriasis [2]. Specifically, keratinocytes and fibroblasts amplify inflammatory responses in psoriasis [3]. Furthermore, studies have also revealed that psoriasis patients have a distinct configuration of skin microbiota, particularly streptococcal, may be one of the triggering factors for psoriasis [4, 5]. The association of psoriasis with several systemic comorbidities, including cardiovascular disease, inflammatory bowel disease, chronic kidney disease, and chronic lung diseases like COPD, asthma has received substantial attention [6–10]. In the past, psoriasis and psoriatic arthritis were not considered diseases closely associated with Interstitial Lung Disease (ILD). But an increasing body of evidence suggests that pulmonary fibrosis may be more concurrent in patients with psoriasis or psoriatic arthritis than in the general population [11–14], particularly in people with severe psoriasis [15].

Idiopathic Pulmonary Fibrosis (IPF) is the most common subtype of ILD characterized by idiopathic and progressive fibrosis, a lung disease with high mortality and limited treatment options [16]. IPF is intricately linked to both genetic predisposition and recurrent environmental exposures. Dysregulated epithelial-fibroblast cross-talk plays a pivotal role, fostering an anomalous and persistent inflammatory response [17]. Recent investigations have advanced the notion that deviations in bacterial burden, diversity, and composition within the pulmonary microbiome of individuals afflicted with IPF could play a significant role in shaping disease pathogenesis and its subsequent progression [18]. Previous meta-analyses have reported microbial dysbiosis and abnormal inflammatory responses in both populations of individuals with IPF and psoriasis [19, 20]. Therefore, psoriasis may potentially be linked to an increased risk of developing IPF in theory. It was once thought that the potential correlation between the two might be attributed to the presence of a rare but recognized complication of immunosuppressants (i.e. Methotrexate, Azathioprine) [21, 22]. Existing evidence from observational studies only suggests a susceptibility of psoriasis patients to concomitant ILD. For instance, Ishikawa et al. reported 21 (4.7%) of 447 patients had the simultaneous existence of psoriasis and interstitial pneumonia [13]. Butt et al. conducted a descriptive study, they retrospectively selected 44 patients with psoriasis or psoriatic arthritis who had clinical evidence of diffuse parenchymal lung disease (DPLD, also known as ILD), where nearly one-third of patients had no prior immunosuppression, and nonspecific interstitial pneumonia (NSIP, a specific type of ILD) and unclassifiable fibrosis were seen in 24 patients (55%) and 8 patients (18%), respectively [14]. In parallel, Wu et al. and Makredes et al. conducted investigations estimating the odds ratios (ORs) for patients with psoriasis or psoriatic arthritis, revealing an elevated prevalence ratio associated with pulmonary fibrosis, ranging from 1.3 (95% confidence interval [CI], 1.1–1.5) to 1.9 (95% CI, 1.2–3.0), indicating that individuals with psoriasis may be at an increased risk of developing pulmonary fibrosis [11, 12]. Although pulmonary fibrosis is a common pathological feature in various subtypes of ILD, including IPF. There is still a lack of direct evidence linking psoriasis to IPF. In addition, both diseases above shared some common risk factors such as smoking, obesity, alcohol consumption, infections, medications, and depression [19]. And these observational studies are often limited to residual confounding and reverse causation. Therefore, the causal relationship between psoriasis and IPF remains uncertain.

Currently, Mendelian Randomization (MR) analysis has been widely used to assess the potential causal relationships between exposures and clinical outcomes [23]. Since random segregation of genetic instruments takes place before the onset of the disease, the independent assortment of genetic polymorphisms ensures the stability of MR analysis, rendering it less susceptible to environmental influences. Compared to traditional observational studies, MR analysis can overcome reverse causality, and minimize the effect of confounding factors [24]. The current study applied MR analysis to examine the causal relationship, the strength of association, and the direction of causality between psoriasis and IPF.

## Materials and methods

### Study design

**Fig. 1:**
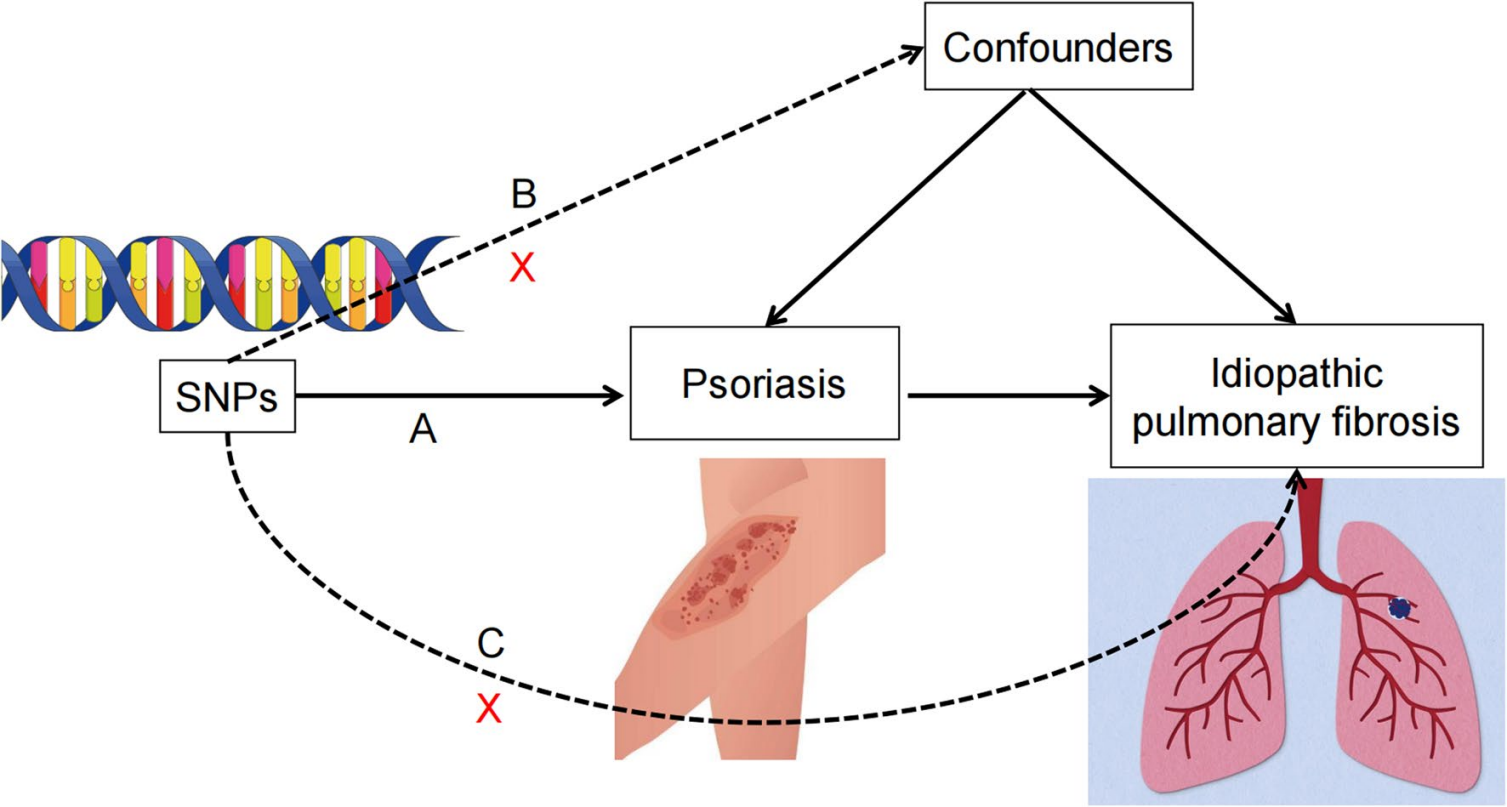
Diagram for key assumptions of MR analyses. **A** SNPs are strongly associated with psoriasis; **B** SNPs are independent of confounders; **C** SNPs must only affect IPF via psoriasis. *SNP* single-nucleotide polymorphism

The schematic diagram of the study design and the three key assumptions of MR are shown in Fig. 1. (A) The single nucleotide polymorphisms (SNPs) are strongly associated with psoriasis. (B) the SNPs are independent of known confounding factors associated with psoriasis. (C) The SNPs only affect idiopathic pulmonary fibrosis (IPF) through psoriasis (Fig. 1) [24].

### Data sources

This is a two-sample MR analysis based on summary statistics from the largest available genome-wide association studies (GWAS), including IEU openGWAS and NealELab. Both GWAS were conducted in people of European ancestry and included both males and females. The GWAS summary statistics to identify genetic risk variants for psoriasis (n = 462,933) were obtained from UK biobank study. The database included 5314 cases with a psoriasis diagnosis plus a control group of 457,619 individuals without psoriasis. Similarly, the GWAS summary statistics for IPF (n = 198,014) were obtained from the FinnGen Biobank Analysis Consortium 2021 as well as 1028 cases and 196,986 controls. The diagnosis of psoriasis and IPF was according to the ICD-10 (International Classification of diseases) criteria. Ethics approval was not required for the current analysis as all included GWAS data are publicly available and had been approved by the corresponding ethical review boards.

### Selection and validation of SNPs

According to the three key assumptions of MR analysis, first, we selected a genome-wide significance level of P < 5 × 10^–8^. Second, a clumping algorithm with a cutoff of r^2^ = 0.001 and kb = 10,000 were used to avoid linkage disequilibrium (LD). When r^2^ > 0.001, the SNP correlated with more SNPs or with a higher P-value was deleted. Third, the F-statistic was calculated to validate the strength of individual SNPs. When F-statistics were greater than 10, SNPs were considered powerful enough to mitigate the influence of potential bias. We also conducted data-harmonization steps to ensure the effects of exposure on the outcome are consistent in terms of the effect allele and subsequent analysis.

### Gene ontology and pathway enrichment analysis

To explore the biological functions and mechanism pathways for these SNPs, the risk SNPs of psoriasis patients were screened for functional enrichment. GO analysis was used to evaluate the degree of enrichment of the SNPs in biological processes, cellular components, and molecular functions. The KEGG pathway is known to grasp the metabolic processes and makes the considerable utility of genomic analysis. The P value < 0.05 was considered as a standard metric for quantifying the top listed pathways.

### Statistical analysis

The primary analysis was conducted using an inverse-variance weighted (IVW) analysis under a random-effects model to combine the instrumental variable-ratio estimates of psoriasis related SNPs [25]. The weighted-median method and MR-Egger method were performed as sensitivity analyses. The weighted-median method can provide valid estimates if over 50% of information comes from valid instrumental variables (IVs) [26]. The MR-Egger method can be used to assess the horizontal pleiotropy of selected IVs [27]. We examined the heterogeneity of the ratio estimators using Cochrane’s Q-value and the MR–Egger intercept among selected IVs. Additionally, a leave one-out sensitivity analysis was conducted to determine whether the overall estimates were disproportionately affected by an individual SNP. All analyses were performed by “TwoSampleMR” packages in R version 4.2.2.

## Results

### Psoriasis related SNPs

**Fig. 2:**
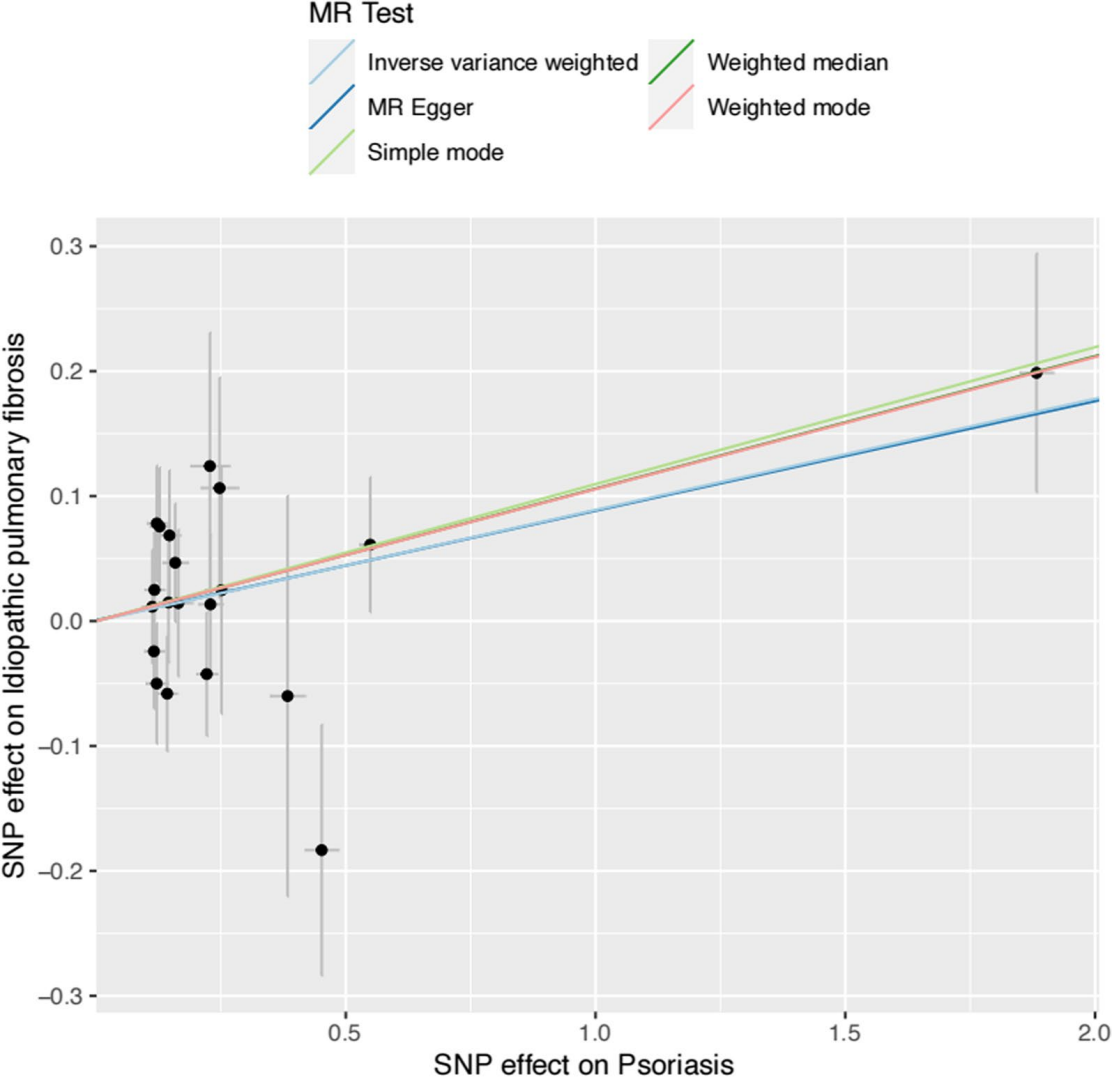
Scatter plot to visualize causal effect of psoriasis on the risk of IPF. The slope of the straight line indicates the magnitude of the causal association. *IVW* indicates inverse-variance weighted, *MR* Mendelian randomization

In this study, we extracted twenty independent genome-wide significant (r^2^ < 0.001, P < 5 × 10 − 8) SNPs as IVs from the GWAS on psoriasis based on a total sample of 5314 psoriasis controls and 457,619 cases. The summary sample was almost entirely of European ancestry. All SNPs used in the MR analysis were robust with F-statistics greater than ten (Table 1). And scatter plot demonstrated the effect of each SNP locus on IPF is shown in Fig. 2. The F statistics of our SNPs were entirely > 30 (range 31.03–3089.49), which indicated that there was not a weak IVs bias for the results.

**Table 1:** The detailed information of 20 SNPs Association with psoriasis

SNP	effect_allele. exposure	Other_allele. exposure	se.exposure	beta.exposure	pos.exposure	pval.exposure
rs11581607	A	G	0.000443	− 0.00259	67,707,690	0.066756
rs4112787	T	C	0.000233	0.001647	1.53E + 08	0.658527
rs842636	A	G	0.000223	− 0.00133	61,091,950	0.435424
rs2111485	G	A	0.000226	0.00138	1.63E + 08	0.606734
rs11135059	A	G	0.000236	− 0.00251	1.59E + 08	0.329065
rs848	C	A	0.000285	0.0018	1.32E + 08	0.816194
rs12188300	T	A	0.00038	0.005126	1.59E + 08	0.093472
rs12189871	T	C	0.000384	0.021364	31,251,924	0.090526
rs28367705	A	G	0.000447	0.007013	31,284,635	0.110929
rs9277937	C	T	0.000376	0.002851	33,184,894	0.096319
rs33980500	T	C	0.00042	0.002807	1.12E + 08	0.074392
rs2735003	G	T	0.000272	− 0.0026	29,808,634	0.207994
rs582757	T	C	0.000247	− 0.00138	1.38E + 08	0.726237
rs13191494	C	G	0.000394	0.004352	32,586,432	0.100222
rs11795343	C	T	0.000226	− 0.00144	32,523,737	0.401328
rs7951925	G	A	0.000229	− 0.00128	1.28E + 08	0.368627
rs8016947	G	T	0.000223	0.001617	35,832,666	0.561824
rs28998802	A	G	0.000322	0.00187	26,124,908	0.140482
rs11085725	T	C	0.000244	− 0.00167	10,462,513	0.292402
rs632376	G	A	0.000224	− 0.00132	48,520,610	0.41992

### Effect of psoriasis on IPF risk

**Fig. 3:**
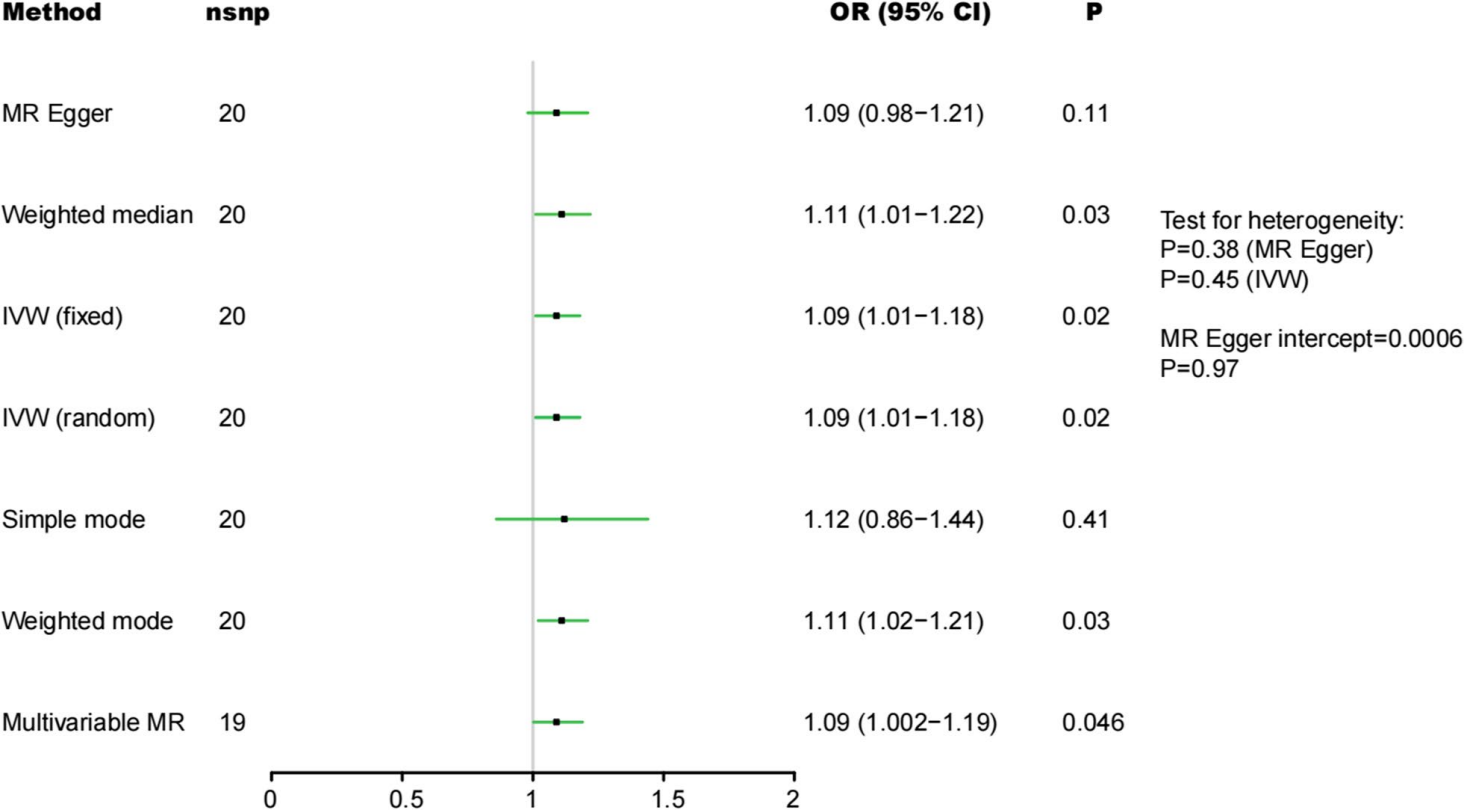
Forest plot to visualize causal effect of psoriasis on the risk of IPF. *IVW* indicates inverse-variance weighted

IVW, weighted median method and MR-Egger regression were used to estimate causal association between psoriasis and the risk of IPF. The IVW analysis showed that the genetically predicted psoriasis increases the risk of IPF, with an odds ratio (OR) of 1.09 (95% confidence interval (CI) 1.01–1.18; P = 0.02). Additionally, the weighted median method (OR, 1.11 (95%CI 1.01–1.22; P = 0.03) and weighted mode (OR, 1.11 (95%CI 1.02–1.21; P = 0.03) had the similar result. MR-Egger regression (OR, 1.09 (95%CI 0.98–1.21; P = 0.11) revealed consistent estimates but of low precision (Fig. 3). In addition, the MR-Egger intercept did not significantly deviate from zero in our study (P = 0.97), suggesting no evidence of ‘horizontal pleiotropy’ or violation of the second MR assumption. Besides, to minimize the influence of common confounding factors, we conducted multi-variable adjustments in the MR analysis. Smoking was included as additional covariates in the regression model to mitigate the confounding effect of smoking on the relationship between psoriasis and idiopathic pulmonary fibrosis risk. Multivariable MR still indicated the similar result (OR, 1.09 (95%CI 1.002–1.19; P = 0.046).

**Fig. 4:**
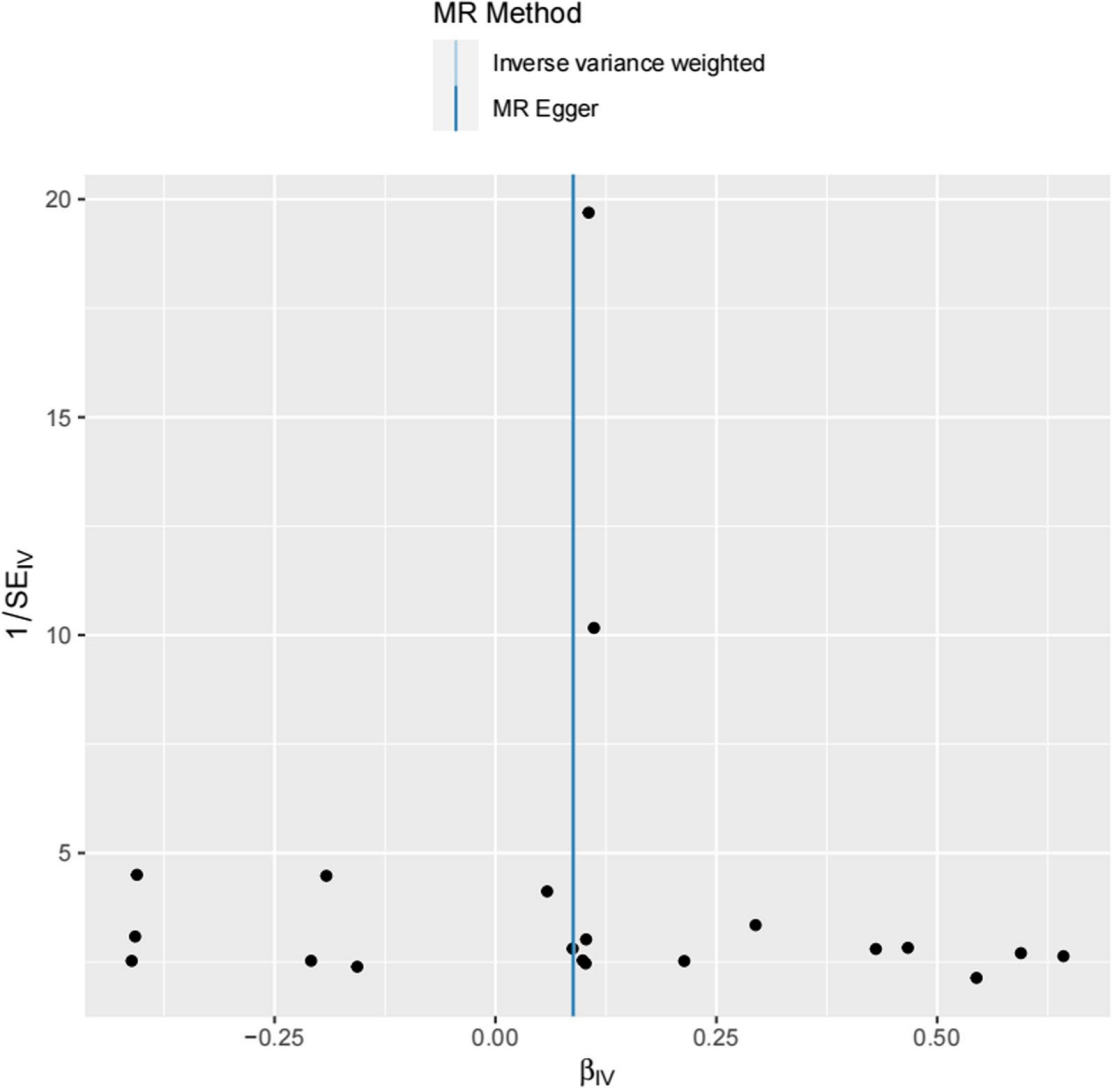
Funnel plots to visualize overall heterogeneity of Mendelian randomization (MR) estimates for the effect of psoriasis on the risk of IPF. *IVW* indicates inverse variance weighted

**Fig. 5:**
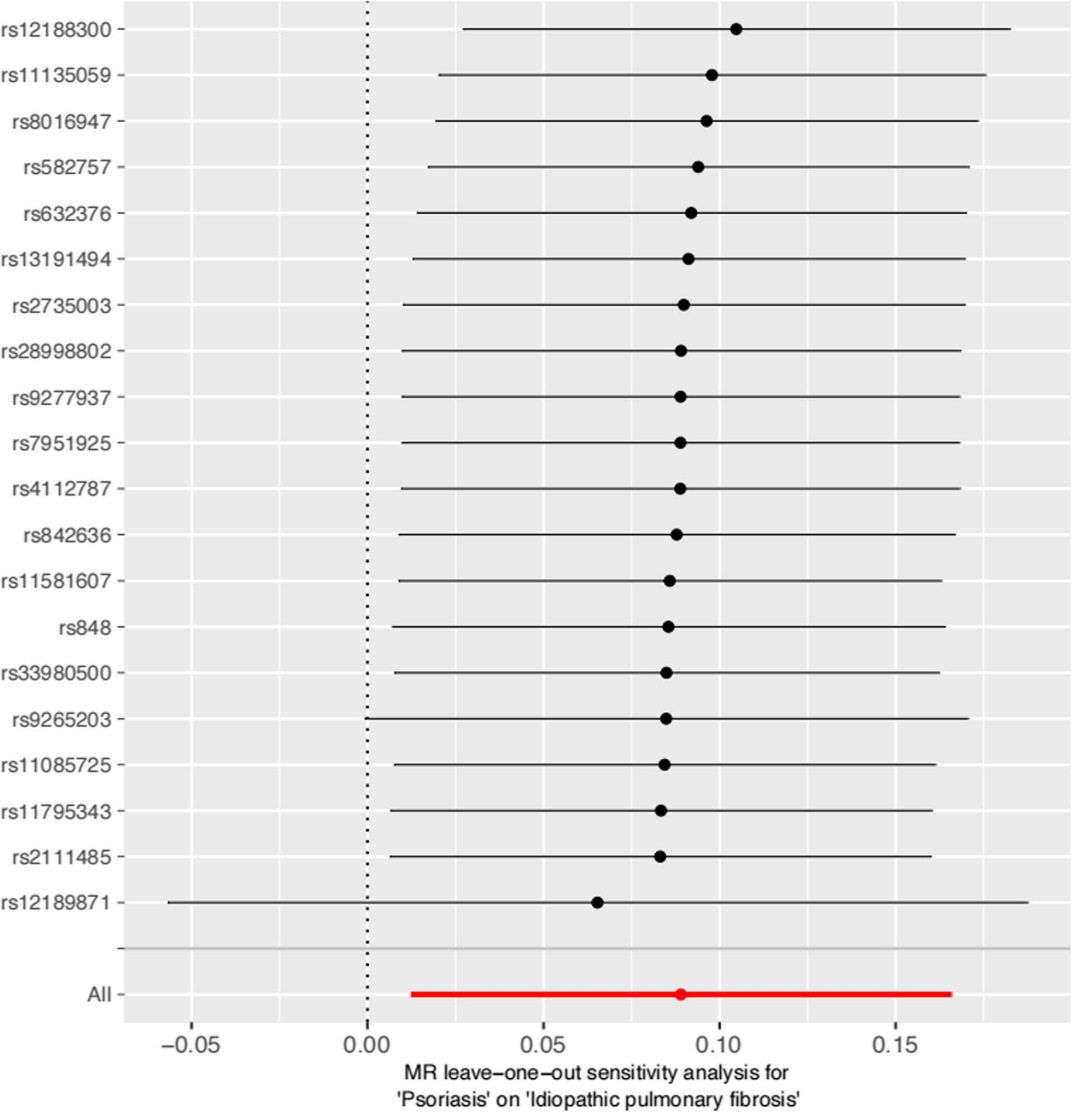
Leave-one-out plot to visualize causal effect of psoriasis on the risk of IPF when leaving one SNP out

Funnel plots indicated no evidence of horizontal pleiotropy (Fig. 4), as it showed that the Wald ratio of each SNP plotted was opposite to their accuracy, while asymmetry indicated directional horizontal gene pleiotropy. The Cochran Q statistics showed that the effect of SNPs included has no significant heterogeneity (Q = 19.12, P = 0.44). Furthermore, Leave-one-out analysis showed that the overall estimates were not disproportionately influenced by any individual variant (Fig. 5).

**Fig. 6:**
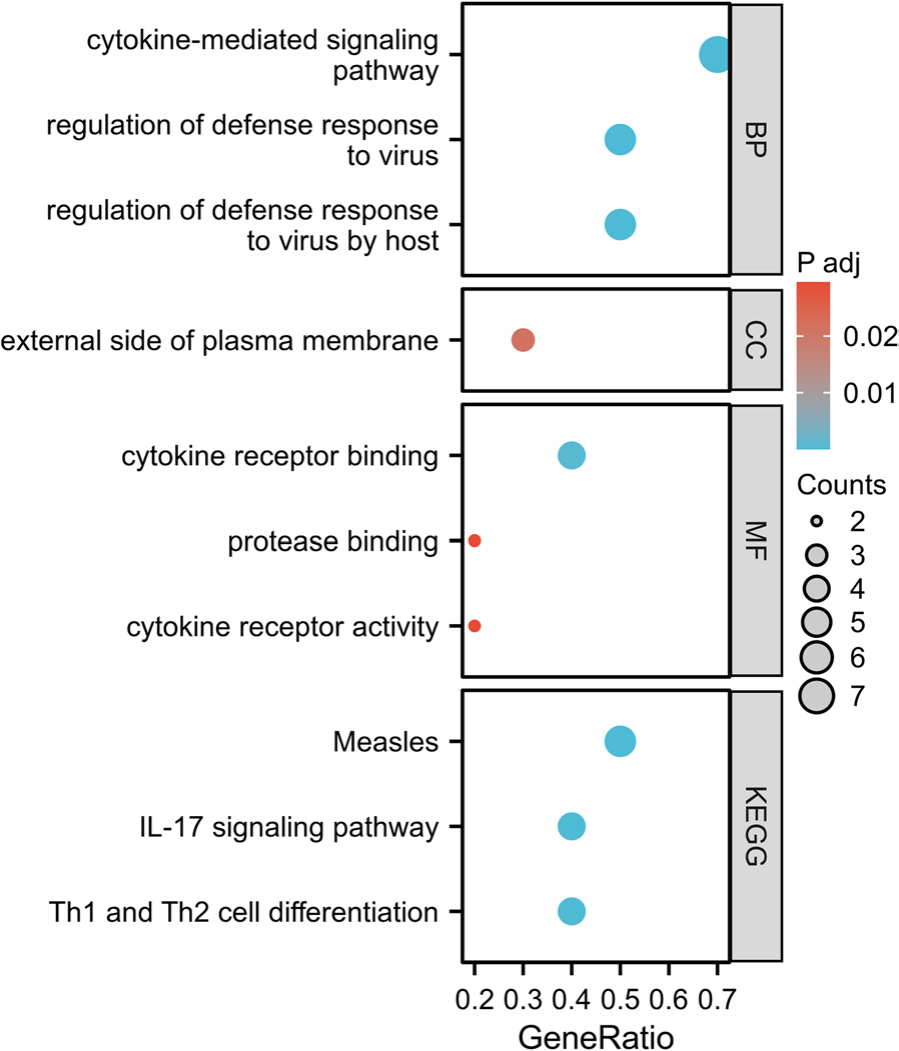
Results of GO and KEGG enrichment analysis using 20 SNPs obtained from MR study. *BP* biological processes, *CC* cellular component analysis, *MF* molecular function analysis

We performed an analysis of Gene Ontology (GO) and Kyoto Encyclopedia of Genes and Genomes (KEGG) using 20 SNPs in this MR study, which showed that cytokine-mediated signaling pathway was the most significant biological pathway and cytokine receptor binding was the top molecular function (Fig. 6).

## Discussion

In the present two-sample MR study, we first set out to assess whether psoriasis, an autoimmune skin disorder, is causally linked to the development of IPF, a condition characterized by a median survival rate of merely 2.5–3.5 years following diagnosis. Our results revealed that the genetic predisposition of psoriasis was associated with an increased risk of IPF (OR, 1.09, 95%CI 1.01–1.18; P = 0.02) in European decent. The positive relationship between two diseases was further verified by sensitive analyses. The results provided compelling insights into the shared genetic architecture, suggesting an increased risk of IPF in psoriasis patients.

In history, psoriasis and ILD were two seemingly disparate diseases. However, as described above, emerging evidence of recent observational studies revealed that ILD were more common in patients with psoriasis than in the general population. Two studies indicated that patients with psoriasis or psoriatic arthritis had an increased prevalence ratio associated with pulmonary fibrosis ranging from 1.3 to 1.9 [11, 12]. A recent observational study in Japan by Kawamoto et.al demonstrated that the incidence of developing interstitial pneumonia in patients with psoriasis was 2%, which is significantly higher than 0.01% in natural condition [28]. Moreover, although ILD was occasionally reported in patients with psoriasis as drug-induced pneumonitis secondary to concomitant use of immunosuppressants in most cases, several cases reported simultaneous existence of psoriasis and ILD with no previous history of immunosuppressant use or biologics [13, 14, 29]. Given that IPF is the most common subtype of ILD, the association between psoriasis and IPF may be underestimated. Concordant with the findings of these observational studies, our study revealed that genetic predisposition to psoriasis was causally associated with a 9.00% increased risk of IPF.

The underlying mechanism explaining how psoriasis increases the risk of IPF remained unknown. Chronic inflammation is postulated as the central etiological factor underlying both conditions. Considerable pro-inflammatory cytokines, including tumor necrosis factor (TNF)-α, type I interferon (IFN), and notably interleukin (IL)-17/23, emanating from cutaneous lesions in psoriasis patients, elicit an aberrant immune response within the pulmonary system via the circulatory system. These same inflammatory cytokines have also been detected in individuals diagnosed with IPF [30, 31], which aligns seamlessly with the SNP instruments we have uncovered. Among the included SNPs, those situated within key loci associated with high levels of T-cell cytokines, such as rs848 within the IL-13 gene and rs11581607 within the IL-23R gene, as well as transcription factors like rs8016947 in the NF-κB gene, demonstrated notable significance in regulating immune responses and inflammation. Furthermore, other variants located in inflammatory cytokine genes, including rs12188300 (IL-12 gene), rs33980500 (TRAF3IP2 gene) and rs582757 (TNFAIP3 gene) were also found to play a role in modulating immune and inflammatory processes. Regarding the relationship between IL-12 and IPF, results are still inconsistent [32]. Some studies suggest that IL-12 may play a certain role in the pathogenesis of IPF, cause IL-12 can stimulate the production of IFN-gamma [33], and the paucity of IFN-gamma may favor the development of progressive fibrosis in IPF [34, 35]. As for the TRAF3IP2 [36] and TNFAIP3 [37] genes, some animal studies have reported pro-fibrotic responses, but a clear association with IPF has not been established. Our findings suggest that the influence of psoriasis on IPF could potentially be elucidated through microbial factors, particularly viral infections. Our GO/KEGG analysis also showed that regulation of defense response to virus was the secondary significant biological pathway, and which included measles, influenza A, hepatitis B virus (HBV), herpes simplex virus (HSV), and Epstein-Barr (EB) virus infection (Fig. 6). The rs34536443 variant may be a bridge linking psoriasis to IPF, rs11795343 is located in the DDX58 gene, which also known as “DExD/H-box helicase 58,” is a gene that encodes a protein called RIG-I (Retinoic acid-inducible gene I), belonging to the DExD/H-box helicase family. RIG-I plays a crucial role in the immune system as a receptor that triggers antiviral immune responses upon viral infection. It plays a pivotal role in safeguarding the body against viral infections and ensuring the homeostasis of the immune system [38]. Disruption or defects in RIG-I function can increase the risk of infections or contribute to the development of autoimmune diseases. DDX58 has been newly identified as a susceptibility gene in psoriasis, it directly causes the production of IL-23 and triggers psoriasis-like skin disease [39]. Certain viruses, such as EBV, CMV, and human herpesvirus (HHV), have been detected in elevated concentrations of individuals with IPF, implicating a potential mechanistic link between latent viral infections and the development of IPF [40, 41]. We assume that DDX58 mediated antiviral immune responses may be one of the potential mechanisms to increase the risk of IPF in psoriasis patients. Furthermore, the rs34536443 variant, located in tyrosine kinase 2 (TYK2) gene, has also been considered to mediate the occurrence of increased risk of IPF. Since TYK2 is a member of the Janus kinase (JAK) family, TYK2 is involved in the intracellular signaling initiated by various cytokines such as IFN, IL-6, IL-12, or IL-23, leading to the phosphorylation of downstream STAT proteins [42, 43]. In psoriasis, the JAK/STAT pathway enhances the inflammatory response by regulating cytokine expression [44]. Likewise, these auto-inflammatory responses may exert a crucial influence on both the initiation and progression of IPF [45].

Therefore, our investigation underscores the potential shared pathways and genetic interplay underlying psoriasis and IPF. These novel genetic insights may have significant implications for understanding the pathogenesis of both diseases and could potentially inform the development of targeted therapies for patients affected by either condition.

However, several limitations to this MR study deserve our attention. First, a measure of disease severity was not available, the proportion of psoriasis cases with different levels of severity will influence the effect size of the outcome. Second, one of the limitations of the UK biobank is the over-estimation of self-reported cases, which can impact the reliability of research conclusions [46]. Indeed, the constraints of coding-based studies encompass inaccuracies in data representation, inherent biases, missing data, and a dearth of clinical details, among other factors. Third, the examined GWASs were primarily conducted in individuals of European ancestry, which might limit the generalization of our findings to other ethnicities, since the incidence of psoriasis varies significantly among different countries. Nonetheless, the European origin reduces the likelihood of population-stratification bias to influence our results. Fourth, genetic factors and environmental factors may have complex interactions, and MR studies may struggle to comprehensively account for these interactions, thus confirming a clear causal association between psoriasis and IPF may require more research.

## Conclusion

In conclusion, this MR analysis provided compelling evidence supporting a causally positive association between psoriasis and IPF. Our results underscore the importance of shared genetic factors in the pathogenesis of these two diseases. Further research into the molecular mechanisms connecting psoriasis and IPF may hold the key to developing targeted therapies and improving patient outcomes in both clinical domains.

## Data Availability

Data available in a publicly accessible repository that does not issue DOIs. Publicly available datasets were analyzed in this study.
